# Improved image quality in GRAPPA-accelerated coronary MRA using an outer volume suppressing 2D-T_2_-Prep

**DOI:** 10.1186/1532-429X-17-S1-P382

**Published:** 2015-02-03

**Authors:** Andrew J Coristine, Jerome Yerly, Matthias Stuber

**Affiliations:** 1 Department of Radiology, University Hospital (CHUV) / University of Lausanne (UNIL), Lausanne, Switzerland; 2 CardioVascular Magnetic Resonance (CVMR), Centre for Biomedical Imaging (CIBM), Lausanne, Switzerland

## Background

Two-dimensional (2D) spatially selective radiofrequency (RF) pulses may be used to excite a restricted volume of tissue. By incorporating a "pencil beam" 2D pulse into a T_2_-Prep module, one may create a "2D-T_2_-Prep" that combines T_2_-weighting with outer volume suppression. This may be of particular benefit to parallel imaging techniques, where artefacts typically originate from residual foldover signal. By suppressing signal from outside the targeted region of interest (ROI), image quality may thus improve. We present numerical simulations, phantom validation, and *in vivo* MRA of the right coronary artery to test this hypothesis.

## Methods

The first RF pulse of an adiabatic T_2_-Prep was replaced with a jinc pulse and spiral gradients. This excites a cylindrical volume. Meanwhile, the final RF pulse remains non-selective; it thus restores the cylinder of T_2_-prepared magnetization, but also rotates outer magnetization into the transverse plane, where it is spoiled. This "2D-T_2_-Prep", and its conventional counterpart, were used prior to normal and GRAPPA-accelerated MRI.

First, a numerical phantom, based on real image data (see below), was used to simulate acceleration factors of R=1..6 with random coil noise. Through repeated simulations, per pixel maps of SNR, noise, and G-factor were predicted for both T_2_-Preps.

Next, the actual phantom, with compartments doped to mimic blood, myocardium, and fat, was scanned 50 times for each acceleration and T_2_-Prep (50x6x2=600 total scans), on a 1.5T Siemens Aera using a gated, 2D gradient echo, 16 channel chest coil, FoV 384x384 (matrix 384x384), 4.0mm slices, TE T_2_-Prep = 40ms, RF angle 20°, and TE/TR/T_acq_=3.4/8.7/69 ms. For each "tissue", an ROI was chosen and the mean SNR_multi_ was calculated.

For *in vivo* experiments, the RCA was imaged in 10 healthy adults, using accelerations of R=1,3, and 6. Parameters were as above, though a volume-targeted 3D sequence was used with 1.5mm reconstructed slices, 24mm volume thickness, water-selective RF excitation pulses of 20°, and TE/TR/T_acq_=5.2/11.6/93.0 ms. Both T_2_-Preps were compared using Soap-Bubble vessel sharpness measurements for each acceleration, and the % differences were calculated.

## Results

**Figure 1:**
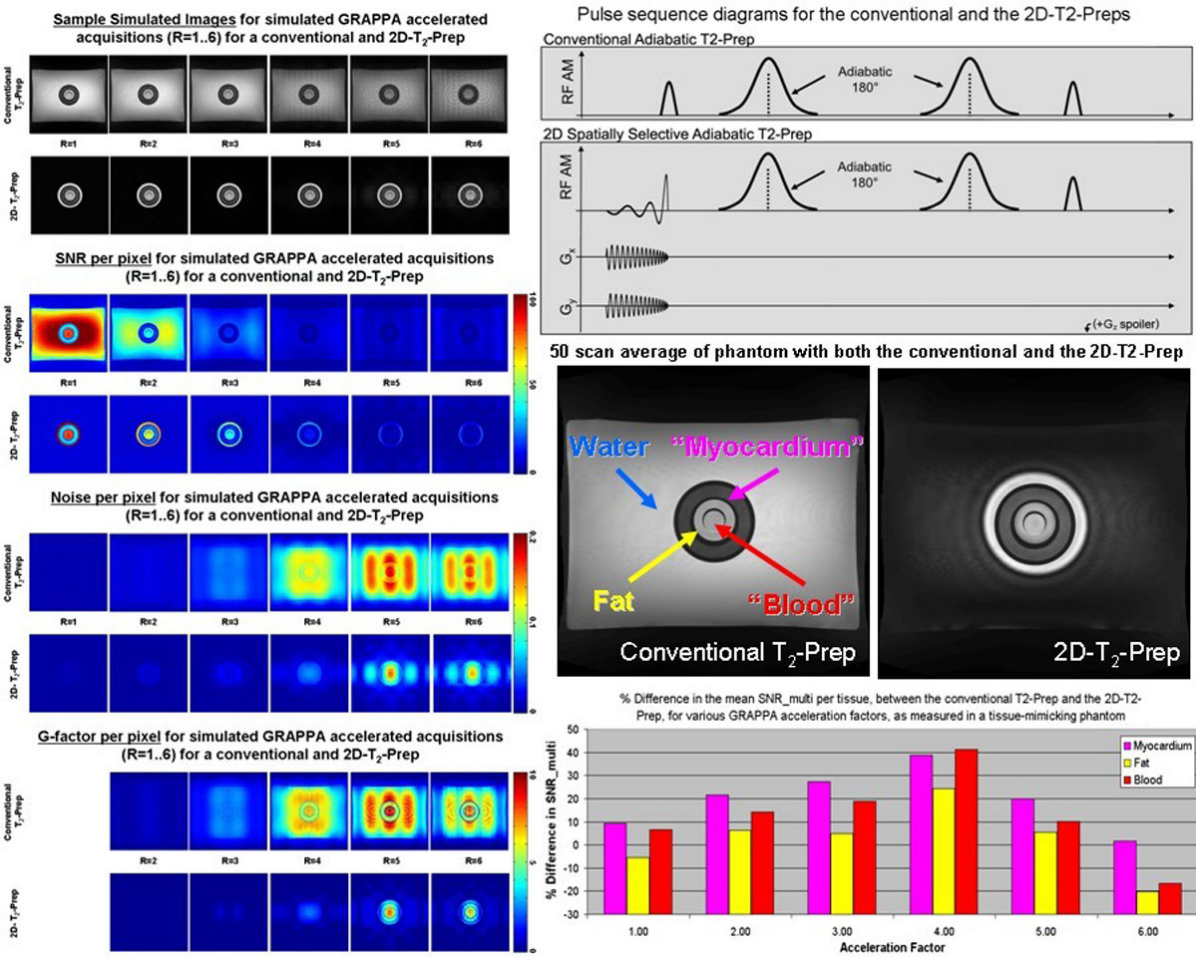
Left side: Simulated GRAPPA-accelerated image acquisitions for R=1,2,3,4,5,6. A numerical phantom, based on real image data, was used to predict the SNR, noise, and G-factor maps for both a conventional and an idealized 2D-T_2_-Prep (pulse sequence diagrams upper right). Right side, middle: 50-scan average of a homebuilt phantom, with compartments doped to mimic fat, blood, and myocardium, using both the conventional and 2D-T_2_-Preps. Bottom right: Mean SNR_multi_ improvement of the 2D-T_2_-Prep over the conventional T_2_-Prep, for various (mimicked) tissue ROIs, at different acceleration factors. The maximal improvement of the 2D-T_2_-Prep occurs when the acceleration factor corresponds to the reduction of excited tissue in the phase encoding (i.e. accelerated) direction.

**Figure 2:**
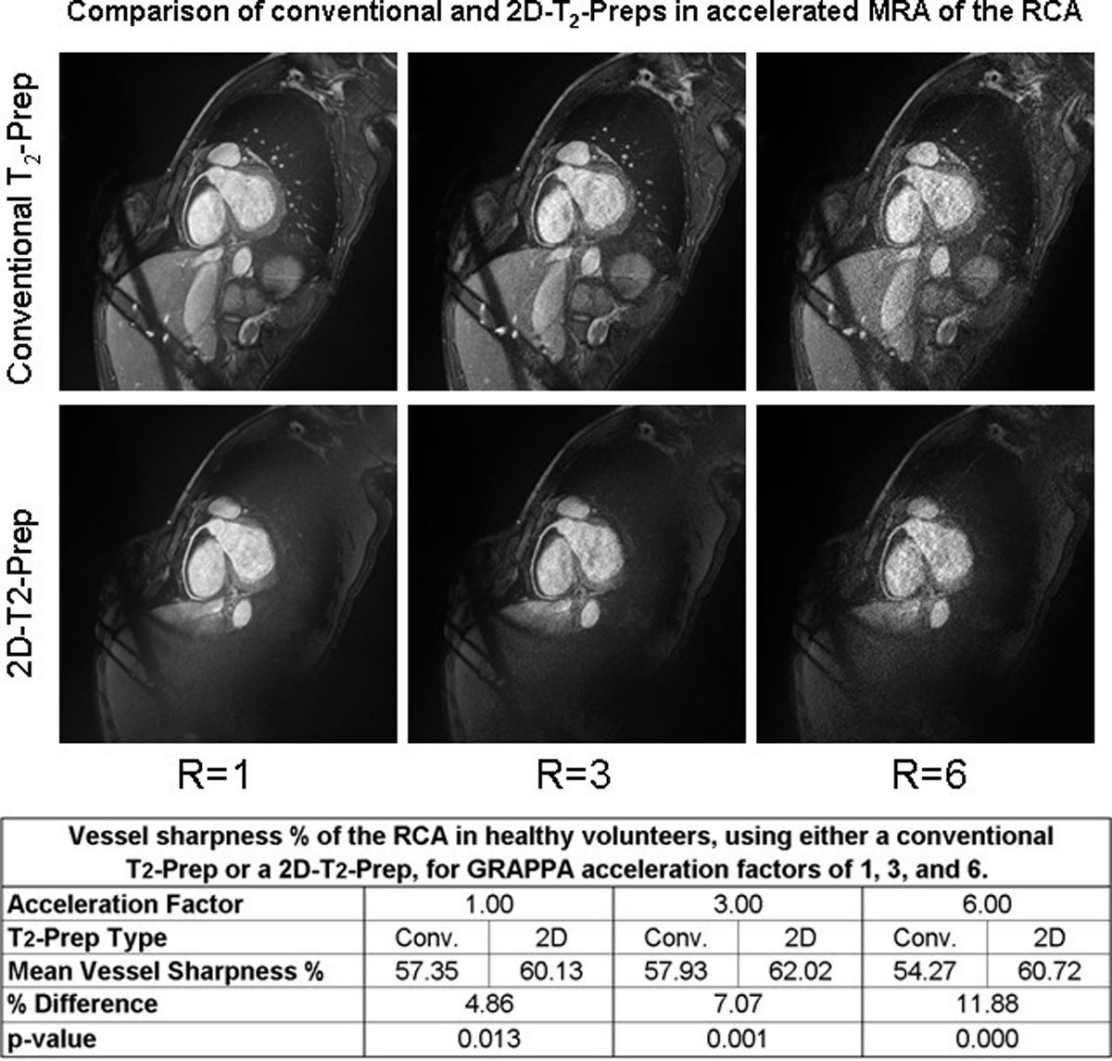
**Sample *in vivo* image acquisitions of the right coronary artery (RCA), using either a conventional or 2D-T_2_-Prep, for GRAPPA acceleration factors of 1,3, and 6.** The mean vessel sharpness improves proportionally more at higher acceleration factors, demonstrating the additional benefit of a 2D-T_2_-Prep.

In simulations, the 2D-T_2_-Prep signficantly improved SNR for accelerated imaging, peaking at a value of R=4. Phantoms also predicted a peak improvement at R=4 (Figure 1, chart). This corresponds to the degree of outer volume suppression of the 2D-T_2_-Prep, specifically the reduction of excited tissue in the phase encoding (i.e. accelerated) direction. For *in vivo* images of the RCA (Figure 2), vessel sharpness improved by a greater % for higher acceleration factors, demonstrating that the 2D-T_2_-Prep especially benefits accelerated coronary MRA.

## Conclusions

Suppressing outer volume signal with a 2D-T_2_-Prep improves image quality particularly well in GRAPPA-accelerated acquisitions in simulations, phantoms, and volunteers, demonstrating that it should be considered when performing accelerated coronary MRA.

## Funding

This work is supported in part by the grant 320030-143923 of the Swiss National Science Foundation.

